# Fabrication of N-Doped Porous Carbon with Micro/Mesoporous Structure from Furfural Residue for Supercapacitors

**DOI:** 10.3390/polym15193976

**Published:** 2023-10-03

**Authors:** Xia Meng, Xiaohui Wang, Wei Li, Fangong Kong, Fengshan Zhang

**Affiliations:** 1 Guangxi Key Laboratory of Clean Pulp & Papermaking and Pollution Control, College of Light Industry and Food Engineering, Guangxi University, Nanning 530004, China; mengxia7184@126.com (X.M.); weili@gxu.edu.cn (W.L.); 2 State Key Laboratory of Biobased Material and Green Papermaking, Qilu University of Technology, Shandong Academy of Sciences, Jinan 250353, China; kfgwsj1566@163.com; 3 Shandong Huatai Paper Co., Ltd. & Shandong Yellow Triangle Biotechnology Industry Research Institute Co., Ltd., Dongying 257335, China

**Keywords:** furfural residue, micro/mesoporous structure, porous carbon, supercapacitor, urea

## Abstract

N-doping is a very useful method to improve the electrochemical performance of porous carbon (PC) materials. In this study, the potential of furfural residue (FR), a solid waste in furfural production, as a precursor to producing PC materials for supercapacitors was highlighted. To obtain an N-doped PC with a high specific surface area (SSA) and hierarchical porous structure, the urea-KOH synergistic activation method was proposed. The obtained FRPCK-Urea showed a high SSA of 1850 m^2^ g^−1^, large pore volume of 0.9973 cm^3^ g^−1^, and interconnected micro/mesoporous structure. Besides, urea can also serve as a nitrogen source, resulting in a high N content of 5.31% in FRPCK-Urea. These properties endow FRPCK-Urea with an excellent capacitance of 222.7 F g^−1^ at 0.5 A g^−1^ in 6 mol L^−1^ KOH aqueous electrolyte in a three-electrode system. The prepared FRPCK-Urea possessed a well capacitance retention at current densities from 0.5 to 20 A g^−1^ (81.90%) and cycle durability (96.43% after 5000 cycles), leading to FRPCK-Urea to be a potential electrode material for supercapacitors. Therefore, this work develops an effective way for the high-valued utilization of FR.

## 1. Introduction

Porous carbon (PC) possesses the advantages of SSA, well-arranged porous structure, good conductivity, and plentiful surface functional groups [1]. Therefore, PC can be widely used in many different fields, such as adsorption [2], electrocatalysis [3], electrode material for supercapacitors [4], and so on. For electrode materials, the hierarchical pore structure of PCs would be extremely important. Micropores can provide high specific capacitance, while the presence of mesopores and macropores is conducive to achieving high power density [5,6]. Wu et al. [7] employed Chinese fir and open-heart fruit shells as raw materials to prepare four types of PCs with similar SSA using steam activation or KOH activation methods to study the influence of pore size on electrochemical performance. The pores in PC prepared by KOH activation were mainly micropores, while a large number of mesopores with pore sizes of 3.5–4.5 nm appear in PC prepared by steam activation. When conducting electrochemical performance tests on the prepared PCs, it was found that the CV curves of the four PC samples were similar, indicating that the SSA is the main factor determining the specific capacitance. However, as the scanning speed increased, the specific capacitance of the PC prepared by KOH activation decreased significantly. This was mainly because of the microporous structure in the PC material prepared via the KOH activation, which would increase the ion diffusion resistance. When the scanning speed is high, the double layer inside the micropores cannot be fully established. Therefore, a high SSA and a hierarchical pore structure are favourable conditions for obtaining better electrochemical performance.

In recent years, because of abundant oxygen functional groups, various biomass and biomass waste were adopted as the raw material of PCs [8]. Furfural residue (FR) is the solid waste in furfural production from corn cobs. There are approximately 23 million tons of FR generated in China per year, and most of FR has not been efficiently managed [3]. The main chemical components of FR are 63 wt% of lignin, 30 wt% of cellulose, and 7% of hemicellulose and other compounds [4]. Lignin is a high molecular weight aromatic polymer with a three-dimensional heterogeneous polycrystalline network structure [9]. Besides, among the main components of biomass, lignin has the highest carbon content, making lignin a potential precursor for PC materials [10]. Cellulose is the most abundant natural polymer on Earth. There are crystalline and amorphous regions in the cellulose structure [9]. Because of the crystalline regions, cellulose is very difficult to hydrolyze even under acidic, enzymatic, and high-temperature conditions [11]. Therefore, the content of cellulose in FR is high. Correa et al. [12] discovered that the SSA of the PC sample prepared by the mixture of cellulose and lignin was higher than that of PC samples prepared by the mixture of cellulose and hemicellulose, hemicellulose and lignin and cellulose, hemicellulose and lignin. Therefore, FR would be a promising precursor for PC because of the high contents of cellulose and lignin. Zhou et al. [2] prepared a P-doped PC from H_3_PO_4_-treated FR, which had a high SSA of 1769.4 m^2^ g^−1^. Guo et al. [4] obtained FR-based PC materials using methanol pretreatment and KOH activation, exhibiting an SSA as large as 1753 m^2^ g^−1^ and a high specific capacitance of 326.1 F g^−1^ at 0.1 A g^−1^.

Recently, it has been generally believed that defects and heteroatom doping/functional group modification have positive impacts on improving the conductivity and wettability of PC [9]. For heteroatom-doped PC materials, nitrogen is the preferred heteroatom. Doping nitrogen into the carbon skeleton can increase the electronic conductivity of PC materials and introduce pseudocapacitance, resulting in improving capacitance [10,11]. Generally, pyrrolic-N and pyridinic-N would enhance the pseudocapacitance of N-doped PC materials by redox reactions [13,14], and graphitic N can increase the electrical conductivity of PC materials by acting as electron donors and/or by attracting protons [15,16,17]. Meanwhile, part of the introduced N atoms can replace C atoms, and then the carbon framework would be destroyed, leading to more active sites for electrolyte ion adsorption [9].

Zhang et al. [18] found that nitrogen doping of PC materials can promote the chemical absorption of Zn^2+^ in zinc ion hybrid capacitors, and greatly improve the conductivity, surface wettability, electrochemical activity, SSA, and ion/electron transport properties. When nitrogen-doped PCs were adopted as the positive electrode material to assemble zinc ion hybrid capacitors, the capacitance can reach up to 177.8 mAh/g, nearly three times that of the undoped PC sample. Therefore, nitrogen-doped PC materials are the preferred choice for electrode materials.

In this work, a novel N-doped PC with high SSA and hierarchical pore structure was prepared using FR by urea-KOH synergistic activation method based on furfural production. The influences of KOH and urea in the PC preparation process were clarified. By this method, urea can work as both a pore-size regulator and nitrogen source to improve the capacitance of the produced N-doped hierarchical PC materials. The excellent hierarchical porous structure and functionalities of the doped N element endowed the obtained PC samples with excellent capacitance, good rate capability, and cycle durability in supercapacitors. Therefore, FR-based N-doped PC is a potential electrode material for supercapacitors.

## 2. Materials and Methods

### 2.1. Materials

Furfural residue (FR) was obtained from Jinan Shengquan Company (Jinan, China), Shandong Province. Urea, KOH, Hydrochloric acid (36% HCl), Sulfuric acid (98%), and ethanol (99.8% CH_3_CH_2_OH) were all purchased from Shanghai Macklin Biochemical Technology Co., Ltd., (Shanghai, China) and were used without being purified. Deionized water was adopted for all experiments.

### 2.2. Methods

#### 2.2.1. Preparation of PCs

PCs were prepared with a urea-assisted KOH activation method by a tubular furnace (OTF-1200X, Hefei Kejing, Hefei, China). Firstly, mixtures of dried FR and KOH with mass ratios of 1:1 and 1:0 were carbonized at 400 °C for 1 h under an N_2_ atmosphere to obtain Pre-FRPCK and Pre-FRPC, respectively. Then, high-temperature carbonizations were performed. The mixtures of Pre-FRPCK and urea (Pre-FRPCK-Urea) or Pre-FRPC and urea (Pre-FRPC-Urea) with mass ratios of 1:1 and 1:0 were heated up to 800 °C at a heating rate of 5 °C min^−1^ for 1 h. After cooling to room temperature, the prepared products were washed with 2 M HCl, absolute ethanol, and distilled water sequentially. Finally, after oven-drying at 105 °C, the PC samples would be obtained. The final PC samples were denoted as FRPC (carbonization without KOH and urea), FRPCK (carbonization with KOH but without urea), FRPC-Urea (carbonization with urea but without KOH), and FRPCK-Urea (carbonization with both KOH and urea).

#### 2.2.2. Characterization of PCs

The morphologies of FRPC, FRPCK, FRPC-Urea, and FRPCK-Urea were evaluated via scanning electron microscopy (SEM). N_2_ isotherms were measured using a Micromeritics ASAP 2020 analyzer to evaluate the porous characters. The SSA and pore size distribution were calculated via the Brunauer-Emmett-Teller and Barrett-Joyner Halenda methods. Raman spectra were obtained via an inVia Reflex Raman spectrophotometer with a 532 nm blue laser beam. FT-IR was obtained by a Bruker FT (ALPHA) infrared spectrometer. X-ray photoelectron spectroscopy (XPS) was used to evaluate the elemental contents and bonding characters of the four as-prepared PC materials. X-ray diffraction (XRD) was adopted to measure the crystal structure of the PC samples by D8 Advance X-ray diffractometer (Bruker, Germany).

#### 2.2.3. Electrochemical Analysis

The electrochemical characteristics of FRPC, FRPCK, FRPC-Urea, and FRPCK-Urea were measured via a three-electrode system. The Pt electrode and Hg/HgO electrode were used for the counter electrode and reference electrode in a 6 mol L^−1^ KOH, respectively [19]. The mixture of PC samples, carbon black, and polyvinylidene fluoride (PVDF) binder with a mass ratio of 8:1:1 was prepared, and then the working electrode slurry can be obtained with N-methyl pyrrolidone (NMP) as the dispersant. Then, the working electrode would be obtained by coating the slurry onto nickel foams and oven-drying at 60 °C. Cyclic voltammetry (CV), galvanostatic charge-discharge (GCD), and electrochemical impedance spectroscopy (EIS) were evaluated on a CHI 660C electrochemical workstation. Meanwhile, the cyclability of the PC sample was also analyzed.

The capacitances would be calculated from the discharge phase of the GCD curves by Equation (1) [20]:

(1) $$C=\frac{I\Delta t}{m\Delta V}$$

where *C* (F g^−1^), *m* (g), and *I* (A) represent the specific capacitance, the mass of PC samples within the working electrode, and the constant current, respectively. Δ*V* (V) represents the voltage window during discharge, and Δ*t* (s) is the discharge time.

## 3. Results and Discussion

### 3.1. Characterization of PCs

**Figure 1:**
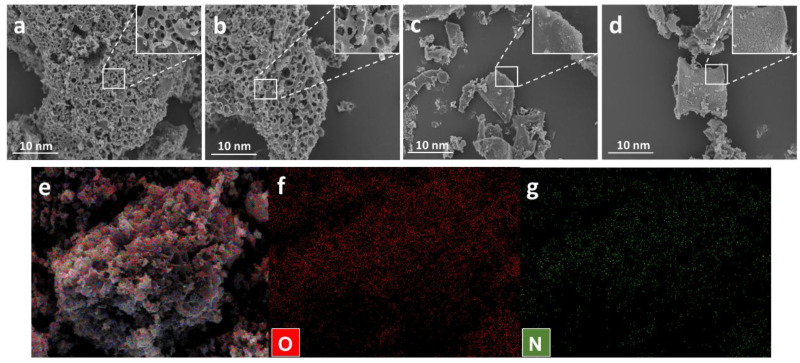
SEM images of FRPCK (**a**), FRPCK-Urea (**b**), FRPC (**c**), and FRPC-Urea (**d**); elemental mapping images of FRPCK-Urea (**e**–**g**).

The morphologies of FRPC, FRPCK, FRPC-Urea, and FRPCK-Urea were measured by SEM, displayed in Figure 1. From the SEM results in Figure 1, it can be discovered that there are obvious differences in the morphology of different PC samples. The surfaces of FRPCK-Urea and FRPCK were rougher than those of FRPC and FRPC-Urea, illustrating that FRPCK and FRPCK-Urea have more sites which is beneficial to energy storage. Meanwhile, FRPCK-Urea and FRPCK possessed well-developed pore structures compared to FRPC and FRPC-Urea, which result from KOH activation. At the same time, the abundant pore structures are interconnected, and these interconnected pore structures can promote the diffusion of ions, which is beneficial to improve electrochemical performance [4]. The distribution of N and O elements in FRPCK-Urea can be detected by EDS, shown in Figure 1e–g. It can be demonstrated that O and N elements are evenly distributed in the carbon material.

**Figure 2:**
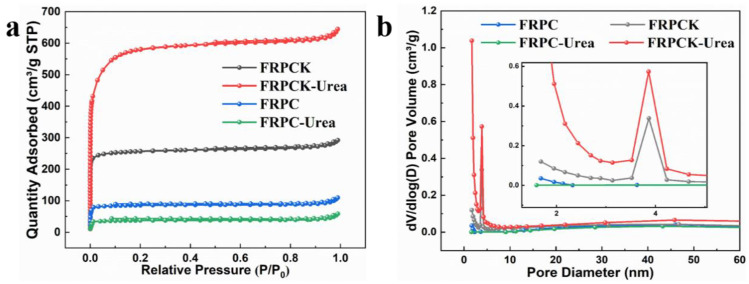
N_2_ adsorption-desorption isotherms (**a**) and pore size distributions (**b**) of FRPC, FRPCK, FRPC-Urea, and FRPCK-Urea.

The porosity properties were analyzed by N_2_ adsorption-desorption, and the results are shown in Figure 2. Also, the porous parameters are exhibited in Table 1. It can be seen from Figure 2a that the N_2_ isotherms of FRPCK, FRPCK-Urea, FRPC, and FRPC-Urea have a clearly sharp upward trend at a relative pressure lower than 0.03 which is a typical type I isotherms feature. The adsorbed volumes of the four samples soared rapidly and saturated quickly, indicating the samples have abundant micropores. From the textural parameters in Table 1, FRPCK-Urea has the highest SSA arriving at 1850 m^2^ g^−1^ distinctly higher than that of FRPCK (824.2 m^2^ g^−1^), illustrating that the addition of urea is very important in increasing the SSA of PC during the synthesis processing. However, the SSA of FRPC-Urea is only 120.7 m^2^ g^−1^, lower than that of FRPC (274.9 m^2^ g^−1^), certificating when the preparation processing of PC without KOH the addition of urea cannot increase the SSA of PCs. Therefore, the combined action of KOH and urea causes PCs to have a high SSA.

From Figure 2b, the as-prepared PC samples are dominated by micropores with a size of 0.5–2 nm. However, mesopores from 2 nm to 5 nm appeared in FRPCK and FRPCK-Urea structures, especially FRPCK-Urea, indicating that KOH and urea have the effect of etching and pore expansion. From the textural parameters in Table 1, comparing FRPC with FRPCK, the mesopores volume ratio of FRPCK-Urea is 25.59%, which is higher than that of FRPCK (19.90%). Similarly, the mesopores volume ratio of FRPC-Urea is 43.70%, which is also higher than that of FRPC (29.03%). These indicate that urea is beneficial to increasing the mesopores volume ratio. Besides, the maximum SSA of mesopores (S_meso_) of 354.1 m^2^ g^−1^ belonged to FRPCK-Urea. Also, the specific surface area of mesopores (V_meso_) exhibited similar trends. FRPC-Urea possessed a higher V_meso_ (0.2541 cm^3^ g^−1^) than those of FRPC (0.0492 cm^3^ g^−1^), FRPC-Urea (0.0397 cm^3^ g^−1^), and FRPCK (0.0899 cm^3^ g^−1^). These results showed that the simultaneous addition of urea and KOH was important in preparing PC samples with micro/mesoporous structures. To a large extent, PC materials with mesopores were identified as promising electrode materials due to accessible pore volumes, highly exposed SSA, and capacious diffusion pathways [21]. As for supercapacitors, these characteristics are beneficial for ions to penetrate into micropores to generate electronic double-layer capacitors [22,23]. Thus, the prepared FRPCK-Urea with interconnected micro/mesoporous structure would be a promising electrode material for supercapacitors.

**Figure 3:**
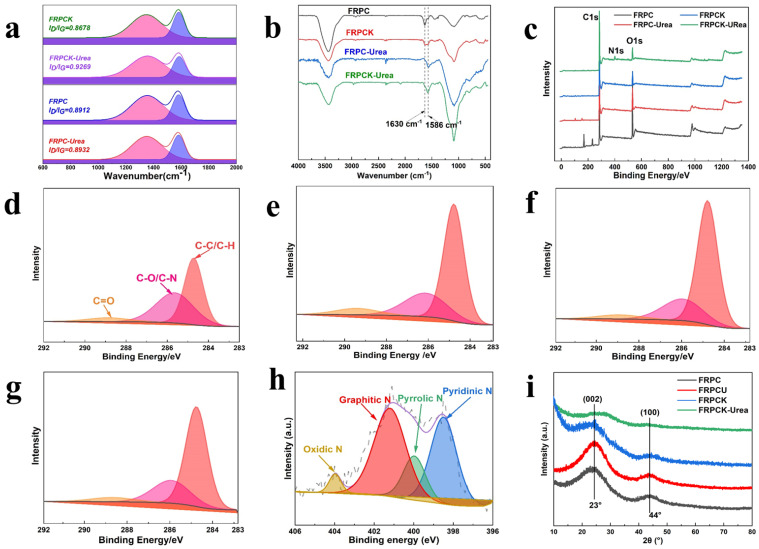
(**a**) Raman spectra; (**b**) FT-IR spectra; (**c**) XPS spectra of FRPC, FRPC-Urea, FRPCK, and FRPCK-Urea; high-resolution C1s spectra of FRPC (**d**), FRPC-Urea (**e**), FRPCK (**f**), and FRPCK-Urea (**g**); (**h**) high-resolution of N1s spectra of FRPCK-Urea; (**i**) XRD patterns of PC samples.

To understand the structure of FRPC, FRPCK, FRPC-Urea, and FRPCK-Urea, the Raman profiles were recorded in Figure 3a. There are two obvious peaks at about 1350 cm^−1^ and 1590 cm^−1^, belonging to the D band and G band [24,25,26]. The intensity ratio of the D-band peak and G-band peak (I_D_/I_G_) were adopted to evaluate the graphitization degree of PC materials [4,27]. The higher the I_D_/I_G_ value is, the more defects and the lower the graphitization degree present [28]. The I_D_/I_G_ values of FRPC, FRPCK, FRPC-Urea, and FRPCK-Urea are 0.8912, 0.8678, 0.8932, and 0.9269. The highest I_D_/I_G_ value of FRPCK-Urea may result from the disorder and amorphous structure. Meanwhile, the higher I_D_/I_G_ value of FRPCK-Urea than that of FRPCK indicated that the urea can work as a nitrogen source and doped N element into a carbon lattice to increase the defect degree. Furthermore, the I_D_/I_G_ value difference of FRPC and FRPC-Urea is not evident, probably because the react processing of urea has changed without KOH.

The chemical properties of these samples were investigated using FT-IR. From Figure 3b, the broad peaks at 3680–3200 cm^−1^ are mainly caused by the overlap of the N–H absorption peak and the –OH absorption peak [29]. The band at about 1630 cm^−1^ was C=O vibration [28,30]. The intensity of the peak decreased after adding urea, indicating the addition of urea would change the group contents in the PC structure. The bank at 1586 cm^−1^ is the C=N vibration [31], and the intensity of the peak increases significantly after the addition of urea, which indirectly indicates that the addition of urea can enhance nitrogen content in the PC structure to improve the electrochemical performance. The bank at 1086 cm^−1^ was C–O–C (symmetric angular deformation of ethers) stretching [29]. These results indicate that N– and O–containing functional groups existed in the as-prepared PC materials.

The surface elemental composition and chemical/electronic states were further evaluated using XPS. From Figure 3c, FRPCK-Urea displayed three peaks at 285.1 eV, 400 eV, and 533.3 eV, ascribed to C1s, N1s, and O1s, respectively [32], while FRPC, FRPCK, FRPC-Urea only showed two obvious peaks (C1s and O1s). This illustrated that the N-doped PC sample was successfully prepared by the urea-assisted KOH activation method, which further proved that urea could work as a nitrogen source to prepare N-doped carbon materials.

It can be seen from Figure 3d–g that there are three peaks at 284.8 eV, 286.1 eV, and 288.6 eV in the high-resolution C1s spectrum, ascribed to C–C/C–H, C–N/C–O, and C=O bond, respectively [29]. Figure 3h shows the high-resolution N1s spectrum of FRPCK-Urea. There were four peaks at 398.2 eV, 400.1 eV, 401.2 eV, and 404.0 eV, ascribed to pyridinic nitrogen, pyrrolic nitrogen, graphitic nitrogen, and oxidic nitrogen, respectively [20,33,34,35]. Detailed information on FRPC, FRPCK, FRPC-Urea, and FRPCK-Urea are exhibited in Table 2. The graphitic C contents of the four samples had some differences. The C–C/C–H content of FRPCK-urea (67.58%) was smaller than that of FRPCK (70.55%), which illustrates that urea can decrease the graphitization degree of carbon materials. However, the C–C/C–H content of FRPC (49.46%) was much smaller than that of FRPC-Urea (64.96%), indicating that the addition of KOH changed the influences of urea on the graphitization degree. Nitrogen can enhance the wettability of PC materials, and therefore increase the accessibility of the electrode surface for improving capacitive capability. As shown in Table 2, the N element content of FRPCK-Urea is 5.313%, which is higher than that of FRPCK (0.7706%), indicating that urea can dope N into carbon materials. However, when preparing PC materials with urea but without KOH, the increase of N content is not evident, which further certifies the reactions of urea have changed without KOH in the PC samples synthesis process. As shown in Figure 3h, the proportions of pyridinic N and graphitic N were relatively high, accounting for 35.99% and 44.89% of the total N content, respectively. Pyrrolic N and oxidized N accounted for 15.55% and 3.572% of the total N content, respectively. The high contents of pyridinic N and graphitic N in FRPCK-Urea would result in good electrochemical performance. The lattice structures of FRPC, FRPCK, FRPC-Urea, and FRPCK-Urea were analyzed using the XRD. From Figure 3i, there are two diffraction peaks at 2θ = 23° and 43°, attributed to the (002) and (100) planes of graphite, respectively, indicating that all PC samples have crystalline and amorphous structures. For FRPCK and FRPCK-Urea, the (002) peaks were weaker than those of FRPC and FRPC-Urea, which was attributed to the presence of extensive porous and defects by KOH activation at high temperatures.

### 3.2. Electrochemical Measurements

**Figure 4:**
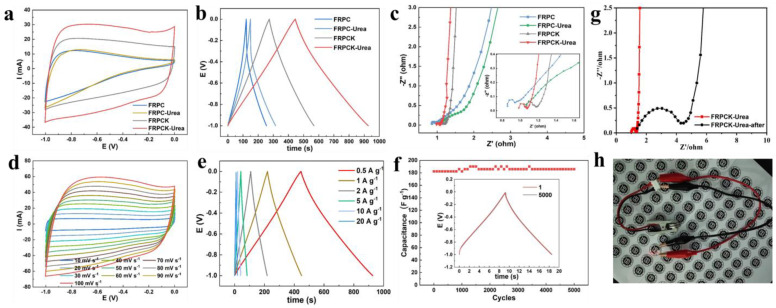
(**a**) CV curves at 50 mV s^−1^; (**b**) GCD curves at 0.5 A g^−1^; (**c**) Nyquist plots; (**d**) CV curves of FRPCK-Urea from 10 mV s^−1^ to 100 mV s^−1^; (**e**) GCD curves of FRPCK-Urea at current density of 0.5–20 A g^−1^. (**f**) cyclic stability test of FRPCK-Urea; (**g**) EIS curves before and after cycling of the FRPCK-Urea; (**h**) Buckle-type supercapacitor lit-emitting diode from the FRPCK-Urea; The measurements were performed in a 1 M KOH aqueous solution using a three-electrode system.

To investigate the capacitive capability of FRPC, FRPCK, FRPC-Urea, and FRPCK-Urea, the electrochemical characteristics were measured using the three-electrode system with 1 M KOH as an aqueous electrolyte. From Figure 4a, the CV curves of FRPCK and FRPCK-Urea exhibited a proximate rectangular at 50 mV·s^−1^, revealing a dominated double-layer capacitance behavior, while CV curves of FRPC and FRPC-Urea have big differences from rectangular because of the low SSA and poor porous properties. The area of the FRPCK-Urea CV curve was the largest, illustrating the highest specific capacitance of FRPCK-Urea, which is due to the high SSA (1850 m^2^ g^−1^) and high N content (5.31%). When comparing FRPC with FRPC-Urea, it can be found that the CV curve areas of the two samples are similar. However, FRPC-Urea possessed a smaller SSA (120.7 m^2^ g^−1^) than that of FRPC (274.9 m^2^ g^−1^). Meanwhile, FRPC-Urea has a higher specific capacitance (77.11 F g^−1^) than that of FRPC (50.23 F g^−1^) calculated from GCD curves (Figure 4b). This indicated that a higher N content in FRPC-Urea (1.8%) than that of FRPC (1.09%) was a key factor influencing specific capacitance. For the GCD curves (Figure 4b), the approximately symmetric triangular shape at 0.5 A g^−1^ was presented for four PC samples, illustrating wonderful and reversible capacitive performances [33,34]. FRPCK-Urea has the longest discharge time, so FRPCK-Urea possessed a high specific capacitance of 222.7 F g^−1^, obviously higher than those of FRPC (50.23 F g^−1^), FRPCK (147.6 F g^−1^), FRPC-Urea (77.11 F g^−1^). When both KOH and urea were added, the specific capacitance of the PC sample can be significantly improved because of the high SSA and high N content, indicating a synergistic effect between KOH and urea.

The EIS (Figure 4c) was evaluated to further analyze the electrochemical characteristics of the as-prepared four samples. In low-frequency region, the curves of FRPCK and FRPCK-Urea were approximately vertical, especially FRPCK-Urea, indicating that FRPCK and FRPCK-Urea (especially FRPCK-Urea) have small electrolyte ion diffusion resistance and good capacitive performance. In the high-frequency region, the semicircle diameter and intercept on the x-axis stand for the charge transfer resistance and solution resistance, respectively [20]. As shown in Figure 4c, FRPC, FRPC-Urea, FRPCK, and FRPCK-Urea all have small semicircle sizes, and the calculated resistance values were 0.1412 Ω, 0.1649 Ω, 0.1861 Ω, and 0.1411 Ω, respectively. Therefore, FRPCK-Urea possessed the smallest resistance value. According to the electric double-layer theory, energy storage is realized by the accumulating charges and ions on the PC surface [20]. High SSA and abundant pores would endow PC materials with outstanding capacitive behavior because high SSA can lead to excellent specific capacitance, and appropriate pore structure is conducive to rapid ion diffusion speed [20]. As shown in Figure 2 and Table 1, FRPCK-Urea possessed a high SSA of 1850 m^2^ g^−1^ with a S_meso_ of 354.1 m^2^ g^−1^. Therefore, FRPCK-Urea had a steep EIS linear curve and a low solution resistance, which makes FRPCK-Urea a promising electrode material. Therefore, the urea-assisted KOH activation method can prepare PC material with excellent capacitive performance.

**Figure 5:**
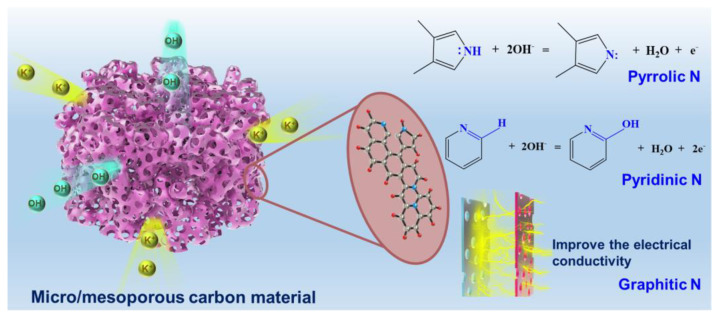
The transport path of K^+^ and OH^−^ and contributions of pyridinic N, pyrrolic N, and graphitic N.

The relationship between electrochemical characteristics and physical-chemical properties of the PC samples were studied. The excellent electrochemical performance of FRPCK-Urea may result from the following reasons: (1) FRPCK-Urea has a high SSA of 1850 m^2^ g^−1^. Also, a well-developed porous structure would improve capacitive properties. FRPCK-Urea possessed a high V_T_ of 0.9973 cm^3^ g^−1^ with a micro/mesoporous structure. The size of K^+^ and OH^−^ in KOH aqueous electrolyte is smaller than the size of most micropores, thus micropores would provide many active centers [36]. Meanwhile, mesopores are beneficial for ions to penetrate into micropores to generate double-layer capacitance [22,23]. (2) the N content (5.31%) of FRPCK-Urea was higher than that of FRPC, FRPC-Urea, and FRPCK. The high levels of pyridinic N (35.99%) and pyrrolic N (15.55%) can increase pseudocapacitance by redox reactions [13,14]. Also, there was a high content of graphitic N (44.89%), which can increase the electrical conductivity of PC materials by acting as electron donors and/or by attracting protons [15,16,17]. The transport path of K^+^ and OH^−^ and the contribution of N atoms are exhibited in Figure 5.

CV curves at scan rates of 10–100 mV s^−1^ and GCD curves at current densities of 0.5–20 A g^−1^ were measured to further study the capacitive performance of FRPCK-Urea. It can be seen from Figure 4d that the rectangular shape of the CV curves of FRPCK-Urea can be well-maintained even at a high scanning rate. From the GCD curves (Figure 4e), it can be calculated that capacitance retention was 81.90%. Meanwhile, the GCD curves remained symmetrical at the current density range of 0.5–20 A g^−1^, illustrating FRPCK-Urea has wonderful reversibility. Additionally, 96.43% capacitance remained after repeatedly charging and discharging at 20 A g^−1^ for 5000 cycles (Figure 4f), illustrating the good working life of the supercapacitors [37]. In addition, the impedance of the sample before and after the cycle was compared and analyzed, and the results are shown in Figure 4g. Obviously, the diameter of the semicircle in the high-frequency region significantly increases after the cycle, and the slope of the low-frequency region deviates from the vertical. This indicates that the impedance increases after cycling, but it is still within a good range, demonstrating that the prepared nitrogen-doped porous carbons as electrode material have good stability. The FRPCK-Urea materials were used to assemble coin cells and light up small light bulbs (Figure 4h). This indicates that the prepared nitrogen-doped porous carbon material has potential application in battery electrodes. Therefore, a high SSA and high N content would endow FRPCK-Urea with a good rate of performance.

## 4. Conclusions

N-doped PC materials were synthesized from FR using a urea-KOH synergistic activation method for high-efficiency electrochemical applications. In the prepared process, the KOH plays a vital role in the etching of porous carbon materials, and the synergistic effect of urea and alkali endowed N-doped PC with a high SSA. The as-obtained FRPCK-Urea with the highest SSA and suitable micro/mesoporous structure delivered an attractive specific capacitance of 222.7 F g^−1^ at 0.5 A g^−1^, excellent rate capability, and cycle durability. In view of the excellent capacitive capability, the prepared FRPCK-Urea would be a potential electrode material in supercapacitors. Therefore, the urea-KOH synergistic activation method was a promising way to prepare micro/mesoporous carbon materials with good electrochemical properties.

## Figures and Tables

**Table 1:** N_2_ adsorption-desorption results of FRPC, FRPCK, FRPC-Urea, and FRPCK-Urea.

Samples	SSA (m^2^ g^−1^)	S_micro_ (m^2^ g^−1^)	S_meso_ (m^2^ g^−1^)	V_T_ (cm^3^ g^−1^)	V_micro_ (cm^3^ g^−1^)	V_meso_ (cm^3^ g^−1^)	V_meso_/V_T_
FRPC	274.9	246.6	28.34	0.1695	0.1203	0.0492	29.03
FRPC-Urea	120.7	104.6	16.03	0.0908	0.0511	0.0397	43.87
FRPCK	824.2	743.8	80.39	0.4520	0.3621	0.0899	19.89
FRPCK-Urea	1850	1496	354.1	0.9973	0.7432	0.2541	25.48

SSA: specific surface area; S_micro_: micropores SSA; S_meso_: mesopores SSA; V_T_: total volume; V_micro_: micropore volume; V_meso_: mesoporous volume; V_meso_/V_T_: mesopores percentage for total pore volume.

**Table 2:** The elemental composition of FRPC, FRPC-Urea, FRPCK, and FRPCK-Urea.

Samples	C	N	O	S	The Content of Carbon Species, %			The Content of Nitrogen Species, %			
					Graphitic C	C–N/C–O	C=O	Pyridinic N	Pyrrolic N	Graphitic N	Oxidic N
FRPC	76.55	1.091	19.01	1.770	49.46	44.11	6.429	−	−	−	−
FRPC-Urea	77.72	1.813	19.28	−	64.96	27.53	7.516	−	−	−	−
FRPCK	81.72	0.7706	14.46	0.014	70.55	24.39	5.061	−	−	−	−
FRPCK-Urea	84.43	5.313	8.445	0.6053	67.58	26.58	5.847	35.99	15.55	44.89	3.572

## Data Availability

The data presented in this study are available on request from the corresponding author.
